# Development of a Neural Network for Target Gas Detection in Interdigitated Electrode Sensor-Based E-Nose Systems

**DOI:** 10.3390/s24165315

**Published:** 2024-08-16

**Authors:** Kadir Kaya, Mehmet Ali Ebeoğlu

**Affiliations:** Department of Electrical-Electronics Engineering, Dumlupınar University, Kutahya 43100, Turkey; mali.ebeoglu@dpu.edu.tr

**Keywords:** e-nose system, interdigitated electrode (IDE) sensor, linear Kalman filter, multilayer backpropagation neural network (ML-BPNN), principal component analysis (PCA)

## Abstract

In this study, a neural network was developed for the detection of acetone, ethanol, chloroform, and air pollutant NO_2_ gases using an Interdigitated Electrode (IDE) sensor-based e-nose system. A bioimpedance spectroscopy (BIS)-based interface circuit was used to measure sensor responses in the e-nose system. The sensor was fed with a sinusoidal voltage at 10 MHz frequency and 0.707 V amplitude. Sensor responses were sampled at 100 Hz frequency and converted to digital data with 16-bit resolution. The highest change in impedance magnitude obtained in the e-nose system against chloroform gas was recorded as 24.86 Ω over a concentration range of 0–11,720 ppm. The highest gas detection sensitivity of the e-nose system was calculated as 0.7825 Ω/ppm against 6.7 ppm NO_2_ gas. Before training with the neural network, data were filtered from noise using Kalman filtering. Principal Component Analysis (PCA) was applied to the improved signal data for dimensionality reduction, separating them from noise and outliers with low variance and non-informative characteristics. The neural network model created is multi-layered and employs the backpropagation algorithm. The Xavier initialization method was used for determining the initial weights of neurons. The neural network successfully classified NO_2_ (6.7 ppm), acetone (1820 ppm), ethanol (1820 ppm), and chloroform (1465 ppm) gases with a test accuracy of 87.16%. The neural network achieved this test accuracy in a training time of 239.54 milliseconds. As sensor sensitivity increases, the detection capability of the neural network also improves.

## 1. Introduction

E-nose systems have become popular in the fields of air pollutant gas detection [1] and air quality monitoring [2] for preventing ecological pollution. In e-nose systems, there are two critical thresholds for the detection of target gases with high precision: the first is the design of new and novel sensor systems, and the second is the development of data processing and compatible machine learning algorithms.

The first threshold in the design of new and novel sensor systems involves the use of traditional e-nose systems with sensor arrays and separation columns. Thus, e-nose systems based on micro gas chromatography (µGC), which enhances the selectivity for target gases, come to the forefront [3]. However, scientific advancements in the production of precise sensors to be integrated with gas chromatography are ongoing. In particular, there are limited studies on integrated µGC applications with IDE sensor-based micro gas chromatography [4].

The second threshold involves various methods and algorithms used in data processing and converting raw data into information. Before data processing, the Moving Average Method [5] and Kalman filter are commonly used to denoise raw sensor data [6,7]. Both methods filter the signal independently of frequency. However, the advantage of the Kalman filter is its ability to denoise without losing information in noisy sensor signals.

The improvement in performance of machine learning algorithms depends on maximizing the variance in the data and ensuring that the data consist of variables that are uncorrelated with each other. In datasets, the removal of non-informative outlier data and the reduction in data dimensions are commonly achieved through Principal Component Analysis (PCA) [8,9,10].

Real-time nonlinear data obtained from sensors are effectively transformed into meaningful information and used in networks with backpropagation algorithms (BPNNs), recursive neural networks (RNNs), and deep convolutional neural networks (DCNNs) [11,12,13,14,15,16].

The foundation of all these machine learning algorithms lies in various mathematical models of neural networks. ANN algorithms are widely used in processing embedded system sensor data in Industry 4.0 [17], predicting and monitoring faults in electrical machinery [18,19], monitoring weather events and environmental impacts [20,21], diagnosing diseases in humans and animals [22], camera observation, distance measurement, and object detection in robotic applications [23], as well as successfully classifying gas mixtures using various gas sensors [24,25,26].

The BPNN (backpropagation neural network) model is particularly preferred for classifying gas sensor data in e-nose systems [27,28]. The main advantage of the BPNN algorithm is its ability to solve nonlinear problems quickly and with high accuracy [29,30]. Its only drawback is the extensive mathematical calculations required for data training. However, modern embedded system technology has mitigated this disadvantage. The implementation of the BPNN algorithm in hardware has been made possible with Field-Programmable Gate Arrays (FPGAs) [24,31]. For all these reasons, the BPNN algorithm and its derivatives are a prestigious choice for target gas detection in e-nose systems. Recent studies indicate that the detection concentration of NO_2_ gas with IDE sensors is above 20 ppm [32]. The detection of NO_2_ gas at lower concentrations is quite favorable compared with other studies on the detection of air pollutants in the literature.

**Figure 1:**
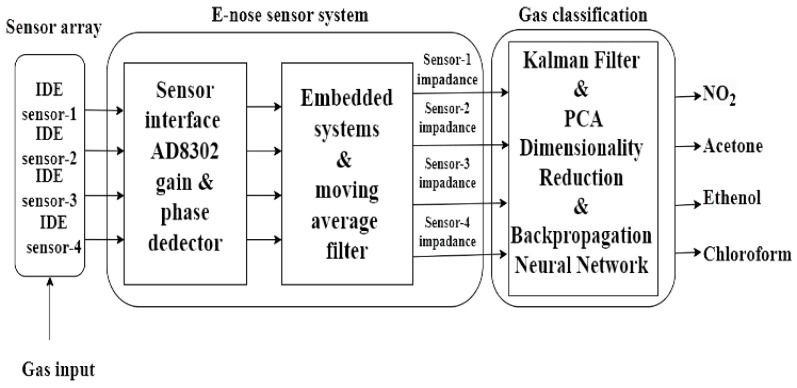
Block diagram for target gas detection.

Figure 1 shows a block diagram summarizing the work carried out on target gas detection with an IDE sensor-based e-nose system. Along with NO_2_ gas, the enhanced BPNN algorithm successfully detected other volatile gases such as acetone, ethanol, and chloroform with high test accuracy.

The development of the e-nose system, details of the enhanced BPNN algorithm, and all experimental work are explained in detail. Initially, the methods used in the e-nose system are focused on. The following Section 3 provides and discusses the results of some laboratory tests to demonstrate the target gas detection capacity of the e-nose system. The conclusion Section 4 emphasizes the potential future impact of this study and the contributions it may offer to other research.

## 2. Materials and Methods

### 2.1. Experimental Setup

**Figure 2:**
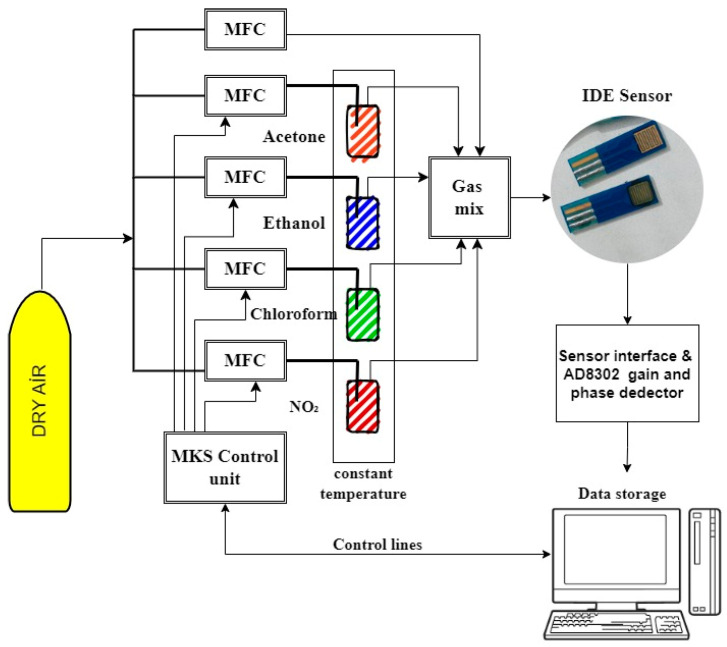
Experiment scheme for IDE sensor-based e-nose system.

In Figure 2, the experimental setup is shown. All experimental gas measurements were conducted at the TUBITAK laboratory under constant conditions of 23 °C and 40% relative humidity. In the experimental studies, pure gases of acetone, ethanol, chloroform, and NO_2_ were used. The flow rates of target gas and air were controlled by calibrated Mass Flow Controllers (MFCs). Dry air was used through a tube to clean the sensors before gas application. Acetone, ethanol, chloroform, and NO_2_ gases were individually added to dry air using bubblers in a temperature-controlled chamber fixed at −15 °C. Desired gas concentrations were achieved using the Antoine equation. Stainless steel tubing was used to maintain the gas temperature constant. The gases entering the gas mixing chamber were applied to the IDE sensor array at a constant flow rate of 200 mL/min. The e-nose system converted measurement results into digital data with a resolution of 16 bits, sampled at a maximum response frequency of 100 Hz, where the maximum response was obtained.

**Figure 3:**
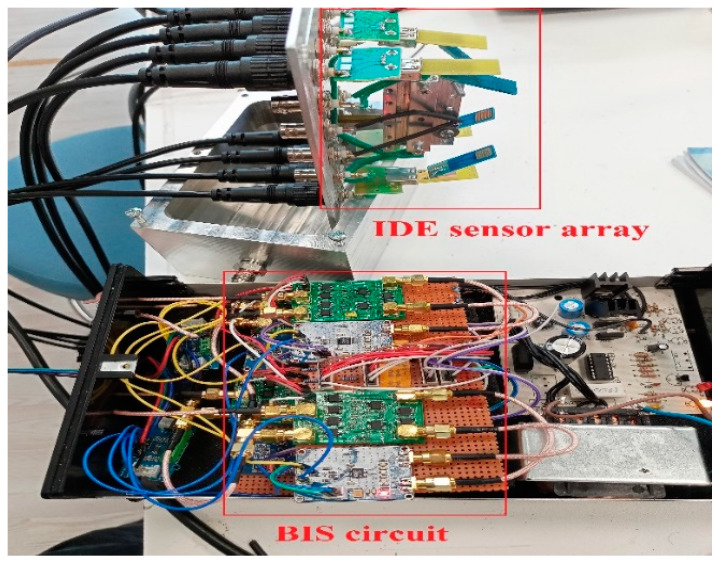
The view of the e-nose sensor system and the sensor array.

In Figure 3, an electronic nose measurement circuit is shown with four arrays of IDE sensors. IDE sensors are organic sensors and are measured with a bioimpedance spectroscopy (BIS)-based interface circuit. The BIS circuit detects changes in impedance magnitude and phase angle occurring in the sensor. The IDE (Interdigitated Electrode) dimensions are as follows: size of IDE: 10 mm × 10 mm; thickness of electrodes (fingers): 20 µm; gap between electrodes: 0.2 mm. The sensor base is silicone and coated with polymer.

### 2.2. Linear Kalman Filter

The Kalman linear filter algorithm has been used to minimize noise in the signals obtained from sensor data without loss of information. Equations (1)–(3) present the mathematical equations used in the algorithm. In the formula, the index *n* is a number starting from zero. The model estimate (*P_n_*), the Kalman gain (*K_n_*), the actual measurement value (*Z_n_*), and the model’s current state estimate (*X_n_*) represent the *n.th* values of the coefficients. Here, $P_{n-1}$ is the previous model estimate, *Q* is the process noise, and *R* is the measurement noise. Here, $P_{n-1}$ denotes the previous model estimate, Q represents process noise, and R denotes measurement noise.

The Kalman filter predicts the current state estimate $X_{n}$ based on a proportional comparison with the real measurement error using the Kalman gain $K_{n}$. According to the Kalman gain $K_{n}$ the difference between the real measurement $Z_{n}$ and the previous model’s predicted value $X_{n-1}$ is added to the last prediction [33,34,35].

(1) $$K_{n}=\frac{\left(P_{n-1}+Q\right)}{{(P}_{n-1}++Q)+R}$$

(2) $$X_{n}=X_{n-1}+K_{n}\times \left(Z_{n}-X_{n-1}\right)$$

(3) $$P_{n}=\left(1-K_{n}\right)\times P_{n-1}$$

Table 1 provides the calculated sensitivities for the target gas measurements of the IDE sensor. In the design of the Kalman filter, the IDE sensor sensitivities were used as a reference for selecting the *R* value for different gases. The *R* value was chosen as $10^{-3}$ for acetone, ethanol, and chloroform gases, and $10^{-1}$ for NO_2_ gas. The process noise value was set to $10^{-4}$.

### 2.3. Principal Component Analysis (PCA)

Before applying PCA, the data were standardized because variables measured in different ppm ranges would otherwise not contribute equally to the analysis and outliers would obscure the results. This process ensures that the data are distributed with a mean of zero (0) and a standard deviation of one (1). The equations used in the algorithm are formulated as follows [36,37].

In Table 2, the covariance matrix obtained from PCA analysis of the sensor array’s response to 0–5460 ppm acetone gas is shown. In Table 2, due to (${IDE}_{i},{IDE}_{k}$)≠ 0 where ${IDE}_{i}$ $\neq {IDE}_{k}$ according to the covariance matrix, there exists a linear association among the sensor data, demonstrating the feasibility of performing PCA analysis on the sensor data. A value greater than zero indicates positive linear correlation, while a negative value indicates negative linear correlation.

Table 3 presents the correlation matrix of the sensor array, standardized from the covariance matrix where ${IDE}_{i}$ $\neq {IDE}_{k}$, providing information on the strength of linear relationships without the need for data normalization [38]. Additionally, the correlation matrix indicates the clusters that should be identified: negative correlation suggests no relationship between the data, indicating separate clustering, while positive correlation suggests clustering together. Since correlation coefficients in the matrix are <0.2, the sensor data exhibit very low linear relationships and high nonlinear variability.

Equations (4) and (5) involve obtaining the eigenvalues and subtracting the eigenvalue space. In these equations, $A_{s}$ represents the covariance matrix of the sensor data set, $\vec{X}$ denotes the sensor data, *λ* stands for the eigenvalues, and *I* represents the identity matrix.

(4) $$A_{s}\vec{X}=\lambda \vec{X}$$

(5) det (λI−A)=0

The eigenvalues of the dataset obtained against 0–5460 ppm acetone gas are found to be 2.2012, 1.4302, 0.2485, and 0.1190 in descending order. The highest eigenvalue indicates the maximum variance or information, guiding us in dimensionality reduction and feature selection processes.

Equation (6) provides the total variance formula, revealing which sensors in the dataset have the highest variance and carry the most information.

(6) $$\sum variance=\frac{\lambda_{n}}{matrix\;diagonal}$$

The 1st principal component represents 55.03% of the total variance, the 2nd principal component represents 35.75% of the total variance, the 3rd principal component represents 6.212% of the total variance, and the 4th principal component represents 3.008% of the total variance.

According to Equation (7), data dimensionality reduction is performed. Here, $\vec{V}$ represents the eigenvector obtained from the eigenvalues, while *i* denotes the number of rows in the sensor dataset, *j* represents the desired reduced dimensionality, and *k* denotes the number of columns in the sensor dataset.

(7) $${\left[S_{reduce}\right]}_{\left(i,j\right)}={\left[S_{normalization}\right]}_{\left(i,k\right)}\times {\left[\vec{V}\right]}_{\left(k,j\right)}$$

The entire dataset comprises 8176 data points (4 × 2044). As a result of dimensionality reduction using PCA, the data column with the lowest variance (3.008%) was reduced. The neural network was trained with 6132 data points (3 × 2044). Overall, 25% of the entire dataset consists of outlier data.

This way, outliers with very little information are removed from the sensor dataset. Throughout the entire classification process of IDE sensors, dimensionality reduction using PCA analysis was employed to transform the 4-dimensional sensor array dataset into a 3-dimensional dataset. Consequently, this led to higher classification performance with fewer mathematical equations and iterations. After dimensionality reduction, the covariance of the new matrix is zero because the eigenvectors are orthogonal to each other, meaning they are perpendicular. There is no relationship covariance between these vectors. Therefore, the covariance matrix of the newly formed PCA-reduced or -transformed data is zero. This eliminates overfitting among the data before classification. With the newly obtained dataset, which has unrelated high variance, classification work has been conducted.

### 2.4. Backpropagation Neural Network (BPNN)

**Figure 4:**
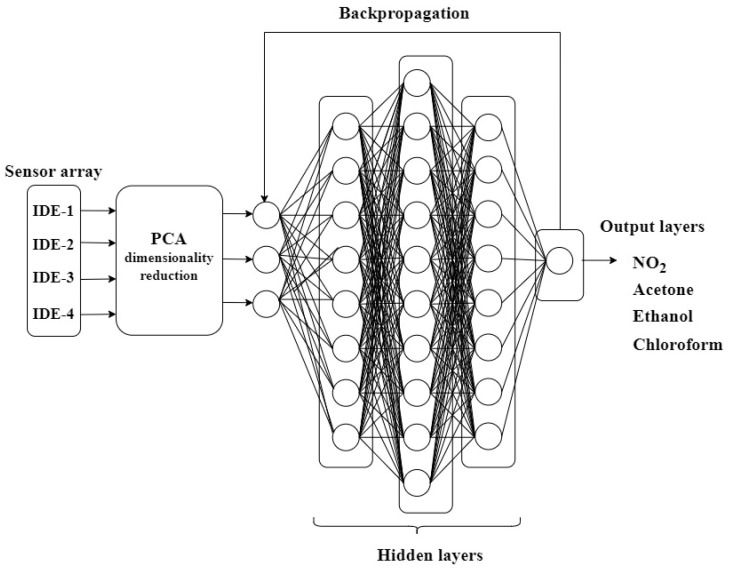
The designed BPNN architecture.

In Figure 4, the neural network architecture that achieved the highest test performance in gas classification is shown. The neural network consists of three hidden layers and one output layer. After dimensionality reduction using PCA, the input dimension of the neural network reduced from 4 to 3. The optimal number of hidden layers that provided the best validation result in the neural network was determined to be 3. The choice of 4 hidden layers is related to the classification of 4 different gases and the high degree of nonlinearity in the data. Since there are no mixed gas data in the neural network design, the network structure was designed with a single output label.

**Figure 5:**
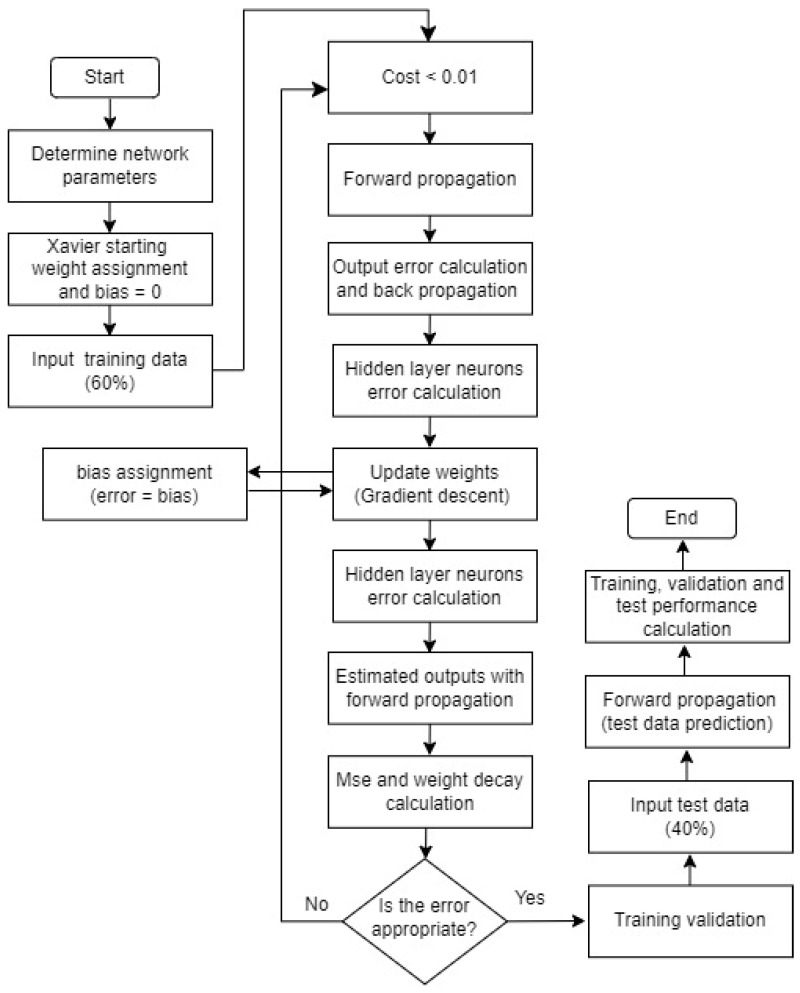
Flow diagram of the BPNN algorithm.

Figure 5 shows the flow diagram of the BPNN algorithm developed for the e-nose system. Unlike traditional BPNN networks, this algorithm is equipped with a deep learning method. The performance of the BPNN has been enhanced by using Xavier weight initialization and controlling the cost calculation with weight decay.

According to Equation (8), the total number of neurons in the hidden layers of the neural network is determined not to exceed 39. Here, $N_{h}$ represents the total number of neurons in the hidden layer, and *n* denotes the number of input neurons [39].

(8) $$N_{h}=(4n^{2}+3)/{(n}^{2}-8)$$

The classification performance of the neural network and the number of neurons in the hidden layer vary according to the sensor sensitivity. To prevent overfitting of the neural network, weight decay has been added to the loss calculation for regularization. Additionally, to test for overfitting between training and testing, and to prevent the network from making predictions based on learned patterns, the data were split into 60% for training and 40% for testing, which the network has never seen before. The experimental groups utilized 6132 data points.

Due to the elimination of overfitting through PCA, no separate validation process was conducted in the neural network training. Sigmoid activation functions were used in both the hidden and output layers, as other activation functions did not achieve the same training and testing performance.

The mathematical equations of the neural network algorithm are provided below [40,41,42]. In training the model, the Xavier initialization method was used for initializing neuron weights to achieve higher performance in target gas detection [43,44].

Equation (9) specifies that neuron weights are initialized using Xavier initialization rather than random initialization. Here, $n_{i}$ represents the number of neurons in the current layer, $n_{j}$ represents the number of neurons in the next layer, and $w_{i}$ denotes the neuron weight in the layers.

Generally, Xavier initialization is used in deep neural networks to ensure that weight gradients across all layers have the same variance. This helps in maintaining the variance in weights throughout all iterations and prevents the vanishing gradient problem. As a result, learning performance improves. The weights are initialized within the range [−1, 1], and bias values are initially set to zero. After each iteration, computed neuron error values are assigned as biases.

(9) $$W_{i}=\left[-\frac{\sqrt{6}}{\sqrt{n_{i}+n_{j}}},\frac{\sqrt{6}}{\sqrt{n_{i}+n_{j}}}\right]$$

Equations (10)–(12) provide the forward propagation equations and the sigmoid activation function used in each layer of the neural network. Here, the *i.th* neuron in the layer represents the *j.th* neuron from the previous layer. Equation (12) gives the mean squared error (MSE) loss function equation.

(10) $$f\left(x\right)=\left(b_{ij}+\sum_{i=1}^{n}x_{ij}w_{ij}\right)$$

(11) $$f\left(act\right)=\frac{1}{1+e^{-x}}$$

(12) $$E=\frac{1}{2}{\left(target-output\right)}^{2}$$

To minimize the error, the gradient descent function is used. The change in error with respect to weights is calculated using the derivative. Equation (13) provides the gradient descent equation for weight updates. Here, $w_{i}$ represents the new weight value, and *α* denotes the learning rate. Throughout all training models, *α* is set to 0.1, which is found to achieve the highest learning efficiency.

(13) $$w_{i}=w_{i-1}-\alpha \times \frac{dE}{dw_{i-1}}$$

In Equation (14), the overall cost calculation between the model prediction and the targets given to the model is performed. Weight decay regularization has been added to the model loss calculation. Here, *λ* denotes the regularization constant, which has been chosen as 0.0001 in the algorithm [45,46].

(14) $$L=\frac{1}{m}\sum_{i=1}^{m}{\sqrt{\left(output-target\right)}}^{2}+0.5*\lambda *\left|w^{2}\right|$$

## 3. Result and Discussion

**Figure 6:**
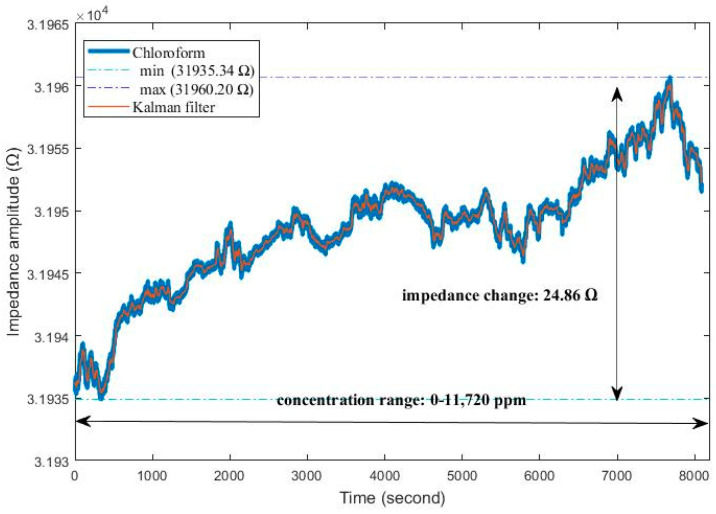
The response of sensor-4 to chloroform gas.

Our research shows that all IDE sensors can be measured at a constant room temperature and a humidity range of 20–80%. Sensor impedance variation is higher at low humidity (20%) and high humidity (80%). At 40% humidity, the sensor impedance has a constant value of 0.8415 Ω on average. Humidity sensitivity is negligible. In Figure 6, the response of Sensor-4 to 0–11,720 ppm chloroform gas is depicted. The sensor impedance change is 24.86 Ω. The sensor was exposed to 600 s of gas followed by 600 s of dry air.

Table 4 presents the sensor sensitivities for different concentration ranges of each gas. Sensor sensitivities were obtained by dividing the impedance change by the gas concentration. The sensor value showing the highest impedance change within the sensor array was taken as the reference. As the gas concentration increases, the sensor impedance change becomes lower. This results in lower sensor sensitivity at higher concentrations. Additionally, decreased sensitivity reduces the accuracy of target gas detection.

**Figure 7:**
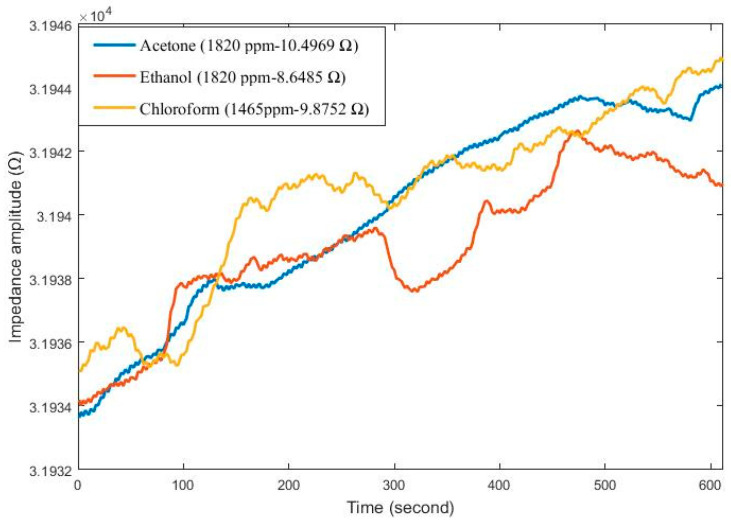
Sensors exhibit the highest impedance change response to acetone, ethanol, and chloroform gases.

Figure 7 depicts the graphical variation in impedance amplitude for sensors within the sensor array responding to acetone, ethanol, and chloroform gases. Sensors were exposed to gas for 600 s after cleaning. Sensor-4 exhibited an impedance change of 10.4969 Ω at 1820 ppm acetone gas, Sensor-3 showed a response of 8.6486 Ω at 1820 ppm ethanol gas, and Sensor-3 responded with an impedance change of 9.8752 Ω at 1465 ppm chloroform gas.

**Figure 8:**
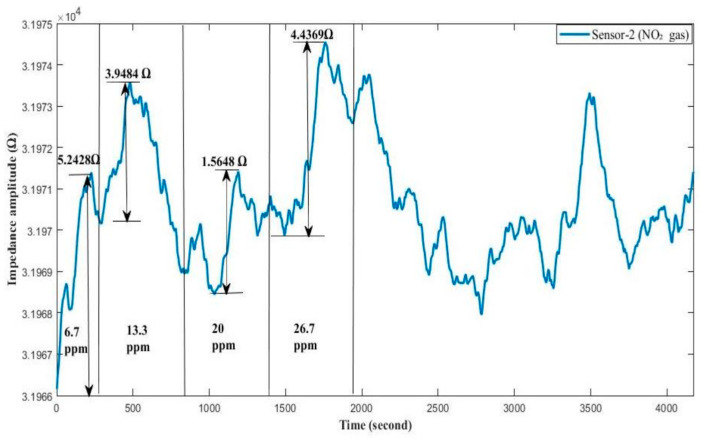
Sensor-2’s impedance change response to NO_2_ gas in the concentration range of 0–46.7 ppm.

In Figure 8, the impedance change graph for Sensor-2 is provided in response to the periodic concentration increase in NO_2_ gas in the range of 0–46.7 ppm. The gas protocol applied to the sensor consists of 300 s of dry air cleaning followed by 300 s of gas exposure. In the classification study, impedance change data were used against NO_2_ gas concentrations where sensor sensitivity and classification performance were highest, specifically at 6.7 and 13.3 ppm NO_2_. After 13.3 ppm of NO_2_ gas, the classification test performance for gas detection fell below 70%.

As seen in Table 5, as sensor sensitivity decreases, neural network classification performance also decreases.

This is because the impedance change is slower relative to the increase in applied gas concentration. The performance criteria include the gradient norm, which is the total magnitude of the vector comprising the errors and biases of neurons in the hidden and output layers. This magnitude is equal to the square root of the sum of squares of these vector values. In the gradient descent algorithm, the goal is to zero out the errors and biases of neurons computed across all layers after backpropagation. Achieving a zero value for this criterion indicates that the neural network has attained maximum performance for the specified mean squared error (MSE) value from training and test data.

**Figure 9:**
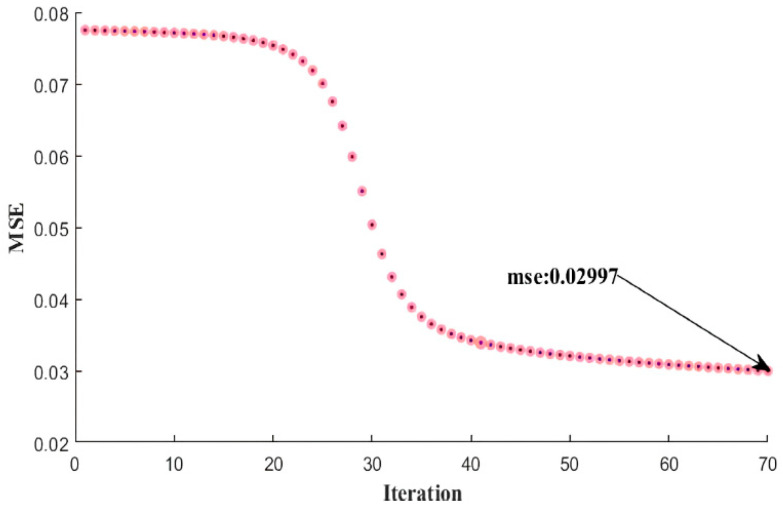
MSE change graph of NO_2_–acetone–ethanol–chloroform gas classification with 87.16% test accuracy.

In Figure 9, the mean squared error (MSE) variation of the neural network algorithm for the classification of 6.7 ppm NO_2_, 1820 ppm acetone, ethanol, and 1465 ppm chloroform gases is shown, with a training accuracy of 84.19% and a test accuracy of 87.16%. The network completed its training with an MSE value of 0.02997 at the 70th iteration in 239.54 milliseconds. For achieving high test accuracy, the variation in MSE with iterations should be linear. The horizontal MSE variation only increases training accuracy without an equivalent increase in test accuracy.

## 4. Conclusions

In this paper, two original studies have been successfully completed and presented to researchers: The first is the design of an e-nose system consisting of an IDE-based sensor array. The IDE sensors used in the e-nose system were custom-made for this study. IDEs are chemically capacitive sensors with no equivalents in the literature. The second is the development of a BPNN algorithm for classifying the high nonlinearity target gas data obtained from the e-nose system. Our neural network model is enhanced with methods used in deep neural networks, differing from the traditional BPNN algorithm. Experimental results have achieved a test accuracy of 87.16% in classifying NO_2_ (6.7 ppm), acetone (1820 ppm), ethanol (1820 ppm), and chloroform (1465 ppm) gases.

The IDE-based e-nose system introduced to the literature, along with the BPNN algorithm developed for gas detection, has yielded practical results for air pollutant gas detection. The developed BPNN model will also serve as a reference for classifying other highly nonlinear gas sensor data.

In future studies, we plan to integrate the IDE sensor array with a micro gas column. This will enable the detection of gas mixtures using a more powerful e-nose system consisting of IDE-based µGC. The performance of the developed BPNN network in the separation of gas mixtures will be demonstrated.

## Figures and Tables

**Table 1:** IDE sensor sensitivities and R value selection.

Gas	Concentration (ppm)	Sensitivity (Ω/ppm)	R (Measurement Noise)
Acetone	1820	5.7 × $10^{-3}$	$10^{-3}$
Ethanol	1820	5.2 × $10^{-3}$	$10^{-3}$
Chloroform	1465	7.4 × $10^{-3}$	$10^{-3}$
NO_2_	6.7	7.825 × $10^{-1}$	$10^{-1}$

**Table 2:** The covariance matrix of the sensor array.

Sensors	Sensor-1	Sensor-2	Sensor-3	Sensor-4
Sensor-1	0.999742	0.246557	−0.64649	−0.67897
Sensor-2	0.246557	0.999742	0.231877	−0.68593
Sensor-3	−0.64649	0.231877	0.999742	0.142882
Sensor-4	−0.67897	−0.68593	0.142882	0.999742

**Table 3:** The correlation matrix of the sensor array.

Sensors	Sensor-1	Sensor-2	Sensor-3	Sensor-4
Sensor-1	1	0.0001	−0.0003	−0.0004
Sensor-2	0.0001	1	0.0001	−0.0005
Sensor-3	−0.0003	0.0001	1	0.00008
Sensor-4	−0.0004	−0.0005	0.00008	1

**Table 4:** Sensor sensitivies (S=impedance/concentration).

Gas	Concentration (ppm)	Sensor Array	Sensor Impedance (ohm)	Sensitivity (Ω/ppm)
NO_2_	6.7	Sensor-2	5.2428	0.7825
NO_2_	13.3	Sensor-1	4.3594	0.3277
Acetone	1820	Sensor-4	10.4969	0.0057
Ethanol	1820	Sensor-3	8.6485	0.0047
Chloroform	1465	Sensor-3	9.8752	0.0067
Acetone	3640	Sensor-2	5.1599	0.0014
Ethanol	3640	Sensor-3	6.5480	0.0017
Chloroform	2930	Sensor-4	5.4573	0.0018

**Table 5:** Results of gas classification using BPNN.

No.	NO_2_ (ppm)	Acetone (ppm) Ethenol (ppm) Chloroform (ppm)	Neuron Number	Training (%)	Test (%)	MSE	Gradient
1	6.7	1820 1820 1465	[8-10-8]	84.19	87.16	0.029970	0.000068
2	13.3	1820 1820 1465	[10-12-14]	89.74	81.70	0.009999	0.000000
3	6.7	3640 3640 2930	[8-10-8]	86.61	76.5	0.019959	0.000067
4	13.3	3640 3640 2930	[12-13-14]	73.28	71.76	0.034974	0.000000

## Data Availability

The data may be made available for sharing.
